# Biological modification of pentosans in wheat B starch wastewater and preparation of a composite film

**DOI:** 10.1186/s12896-022-00734-w

**Published:** 2022-01-17

**Authors:** Piwu Li, Fei Zhao, Xiaofeng Wei, Xiangling Tao, Feng Ding

**Affiliations:** 1 School of Bioengineering, Qilu University of Technology, Shandong Academy of Sciences, Daxue Road 3501, Changqing District, Jinan, 250353 People’s Republic of China; 2 State Key Laboratory of Biobased Material and Green Papermaking (LBMP), Qilu University of Technology, Shandong Academy of Sciences, Jinan, 250353 People’s Republic of China

**Keywords:** Biological modification, Chitosan, Composite film, Wheat pentosan

## Abstract

**Background:**

Petrochemical resources are becoming increasingly scarce, and petroleum-based plastic materials adversely impact the environment. Thus, replacement of petroleum-based materials with new and effective renewable materials is urgently required.

**Results:**

In this study, a wheat pentosan-degrading bacterium (MXT-1) was isolated from wheat-processing plant wastewater. The MXT-1 strain was identified using molecular biology techniques. The degradation characteristics of the bacteria in wheat pentosan were analyzed. The results show that wheat pentosan was effectively degraded by bacteria. The molecular weight of fermented wheat pentosan decreased from 1730 to 257 kDa. The pentosan before and after the biological modification was mixed with chitosan to prepare a composite film. After fermentation, the water-vapor permeability of the wheat pentosan film decreased from 0.2769 to 0.1286 g mm (m^2^ h KPa)^−1^. Results obtained from the Fourier-transformed infrared experiments demonstrate that the wave number of the hydroxyl-stretching vibration peak of the membrane material decreased, and the width of the peak widened. The diffraction peak of the film shifted to the higher 2θ, as seen using X-ray diffraction. The cross-section of the modified composite membrane was observed via scanning electron microscopy, which revealed that the structure was denser; however, no detectable phase separation was observed. These results may indicate improved molecular compatibility between wheat pentosan and chitosan and stronger hydrogen bonding between the molecules. Given the increased number of short-chain wheat pentosan molecules, although the tensile strength of the film decreased, its flexibility increased after fermentation modification.

**Conclusion:**

The findings of this study established that the physical properties of polysaccharide films can be improved using strain MXT-1 to ferment and modify wheat pentosan. The compatibility and synergy between pentosan and chitosan molecules was substantially enhanced, and hydrogen bonding was strengthened after biological modification. Therefore, modified pentosan film could be a potential candidate material for edible packaging films.

## Background

Approximately 30% of all plastic products worldwide are used as packaging materials [1]. However, petrochemical resources, the raw material of petroleum-based plastic products, are becoming increasingly scarce. Furthermore, petroleum-based plastic materials adversely impact the environment owing to their toxicity and non-degradability. Thus, petroleum-based materials should be replaced with new and effective renewable materials. Xylan-type hemicellulose from agricultural and forestry crops has been studied by many scholars as a biodegradable, edible film material. Currently, crop wastes such as rye flour, oats, and arabinoxylan in wheat bran have been studied extensively with the objective of preparing film materials [2]. In addition, arabinoxylan extracted from wheat flour has been used to prepare edible films [3].

Pentosans are non-starch polysaccharides and a type of hemicellulose that exists in the cortexes of different grains. In addition to starch and gluten, wheat is also rich in non-starch polysaccharides, most of which are pentosans [4]. Industrial production of wheat gluten generates 2.4 × 10^7^ tons of starch wastewater every year. Moreover, owing to its high viscosity, wastewater is difficult to treat using conventional sewage-treatment methods [5]. Wheat starch-processing wastewater has potential as a rich source of pentosans. Furthermore, the recycling of wastewater can also improve the economic efficiency of factories and reduce pressure on waste water treatment facilities [6].

Data from previous studies have shown that pentosan has beneficial effects on health [7, 8]. For example, arabinoxylan offers many benefits for human health, including cholesterol-lowering activity, anti-type II diabetes effects, mineral absorption enhancement, stool-bulking effects, and prebiotic benefits [9]. Moreover, gluten-free bread made with pentosan can be used as an alternative bread for people who suffer from celiac disease [10].

Pentosans have attracted considerable attention as sources of hemicellulose. In recent years, many studies have evaluated the applications of pentosans as film materials [11]. Polysaccharide films used for food packaging can isolate the food from ambient oxygen, prevent moisture from evaporating, and preserve aroma and flavor. However, the application range of polysaccharide films is limited by their poor mechanical strength, stability, and toughness. Glucuronic acid/xylan films can be modified by plasticization. Increasing the plasticity of the films by 50% improves the ductility of a film and ensures its flexibility [12]. Acetylized-arabinoxylan and bleach treatment can improve the moisture-barrier properties of the hemicellulose film [13]. Mixed films containing chitosan and xylan have good hydrophobic properties, tensile strength, and other mechanical properties [14]. In addition, the tensile strength of high-molecular-weight arabinoxylan films is significantly lower than that of low-molecular-weight films [15].

The content of pentosans in wheat flour is approximately 2%, and the content of water-soluble pentosans is approximately 0.5% [16]. To increase the utility of water-insoluble pentosans and improve the mechanical strength of polysaccharide films, the molecular weight of water-insoluble pentosans should be reduced [15].

Biological modification presents greater advantages over the other modification methods. First, organic matter can be used to treat industrial wastewater, which can produce enzymes that degrade wheat pentosan and at the same time improve the efficiency of wastewater utilization. Second, during the biological modification process, microbial fermentation produces a complex array of enzymes that is more efficient than pure enzymatic modification. Moreover, the biological modification process of chitosan does not involve the harsh conditions involved in chemical and physical modification processes. Therefore, biological modification is gaining attention and is expected to become an important method for chitosan modification [17]. In addition, in the process of biodegradation of lignocellulose, a single enzyme often cannot degrade the dense structure of lignocellulose, and therein lies the advantage of biological modification as it involves multiple enzymes produced by microorganisms. The synergistic action of the arsenal of diverse enzymes ensures improved degradation and hence, is more advantageous [18].

In this study, wheat pentosan-degrading bacteria were selected from wheat starch wastewater and used to control the relative molecular weights of pentosans. Water-insoluble pentosans were converted into water-soluble pentosans, and improvements in the processability of the modified pentosan chitosan film were analyzed.

## Results and discussion

### Separation and screening of strains

**Fig. 1:**
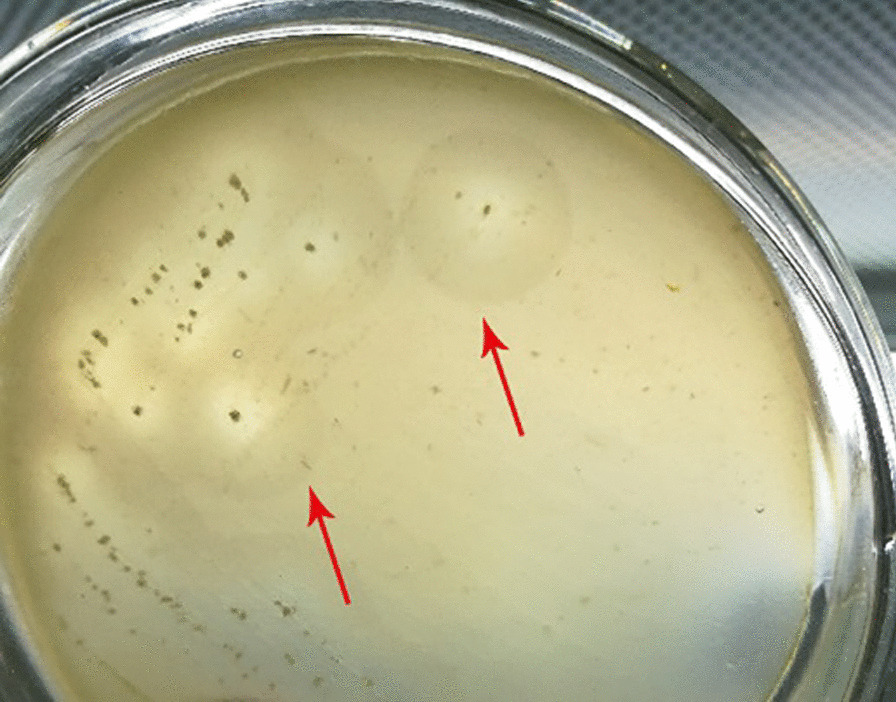
Screened target strains

A single colony for each strain was picked from a separation plate, transferred to a screening plate, and incubated at 37 °C for 2 d. After the pentosan degraded, a transparent circle formed. The screening results are shown in Fig. 1. Wheat pentosan was used as the only carbon source in the screening medium, which enabled screening for strains that use wheat pentosan.

**Fig. 2:**
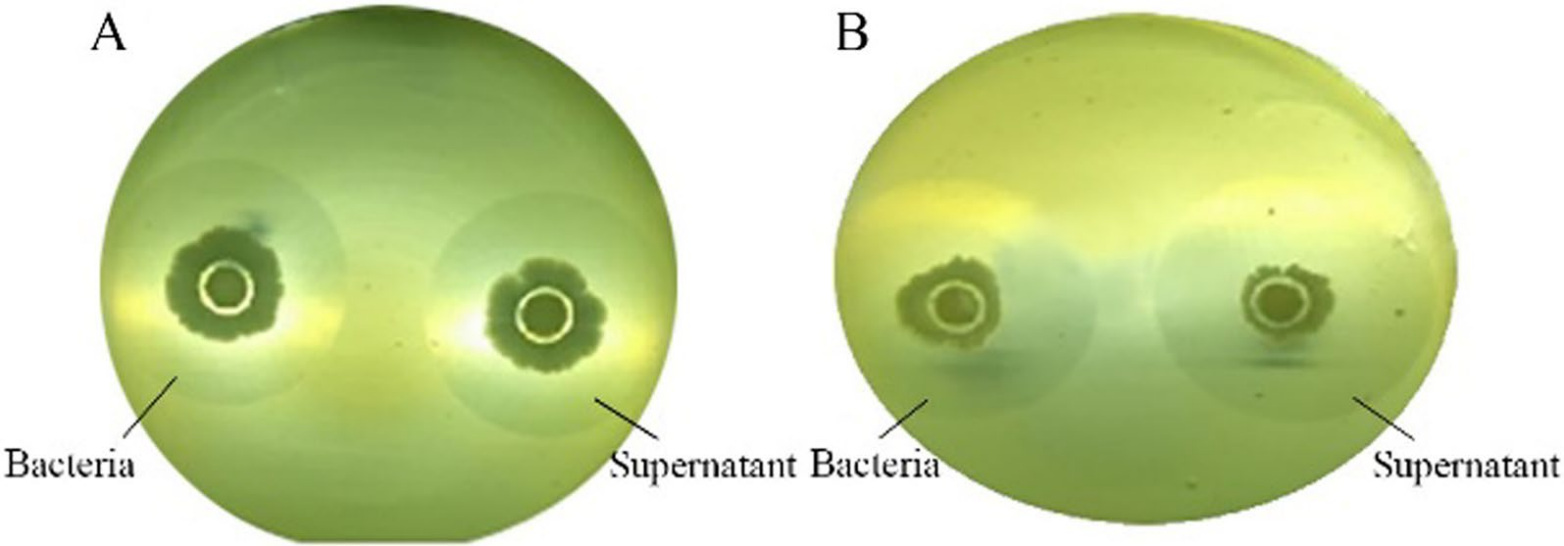
Cultivation of strains and supernatants in different carbon sources. **A** Glucose as a carbon source. **B** Wheat pentosan as a carbon source

As shown in Fig. 2, the strains were cultured on media containing different carbon sources that both produce enzymes capable of degrading wheat pentosan, indicating that the enzyme produced by the bacteria was not inducible. In addition, the supernatants cultured with different carbon sources also showed enzyme activity. These results suggest that the related bacterial enzymes were produced in secreted form.

### Degradation activity of target strains on wheat pentosan

**Fig. 3:**
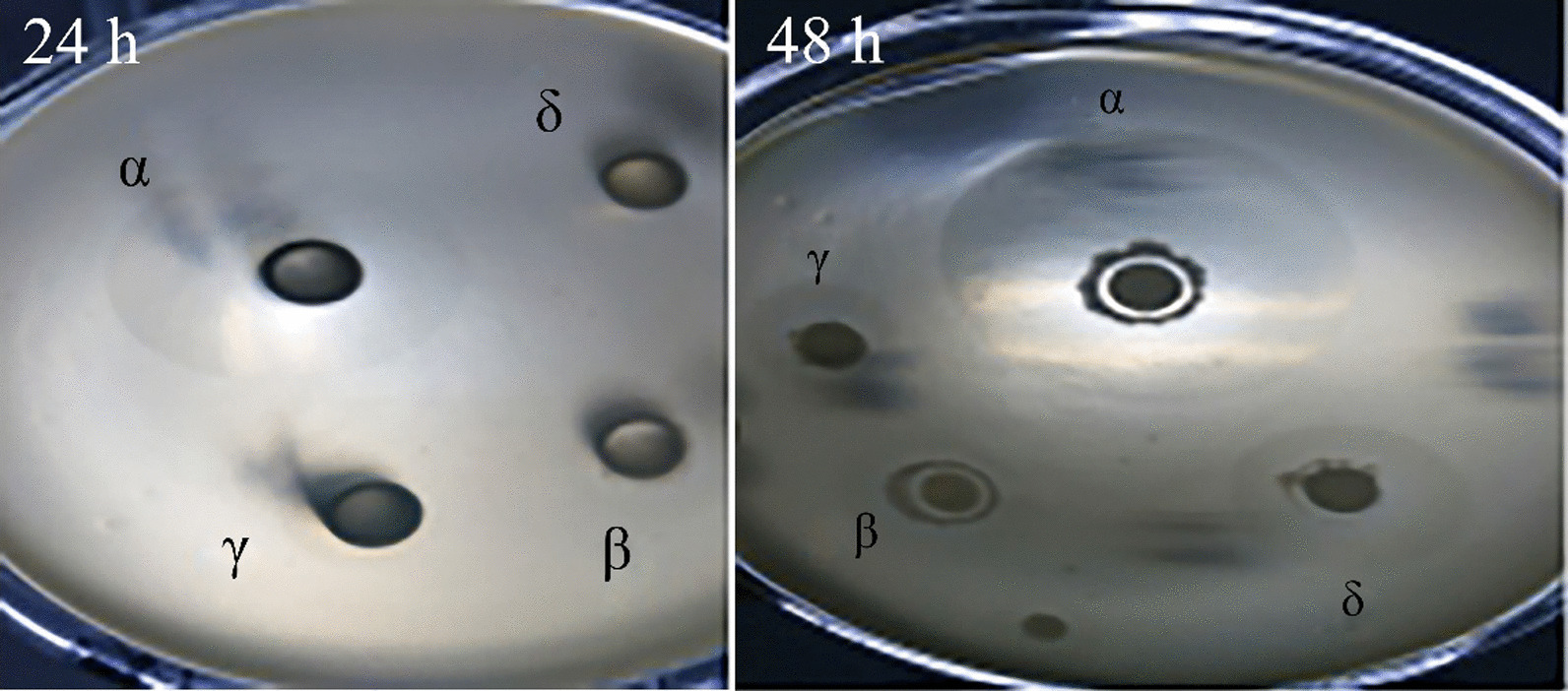
Degradation of pentosan by different strains cultured for 24 and 48 h. (α) Screened strain, (β) *B. subtili*s W800n, (γ) *B. licheniformis*, (δ) *Pichia pastoris*

The screened strains, *Pichia pastoris* cells, *B. subtilis* cells, and *B. licheniformis* cells were simultaneously inoculated into a screening plate. After 24 h of culture, only the α-strains showed transparent circles. After 48 h of culture, the transparent circles surrounding the α-strains became more prominent. As shown in Fig. 3, wheat pentosan was barely degraded by the other strains. This observation validates that the obtained strain depicts the highest wheat pentosan degradation capacity. Ruiz et al*.* also used similar experiments to assess the degradation of xylan and cellulose by the Bacillus strain screened from soil [19].

### Bacterial identification of the target strain

The bacterial 16S rDNA gene was sequenced, and the results showed that the 16S rDNA gene was 1452 base pairs long, consistent with the results of electrophoresis.

**Fig. 4:**
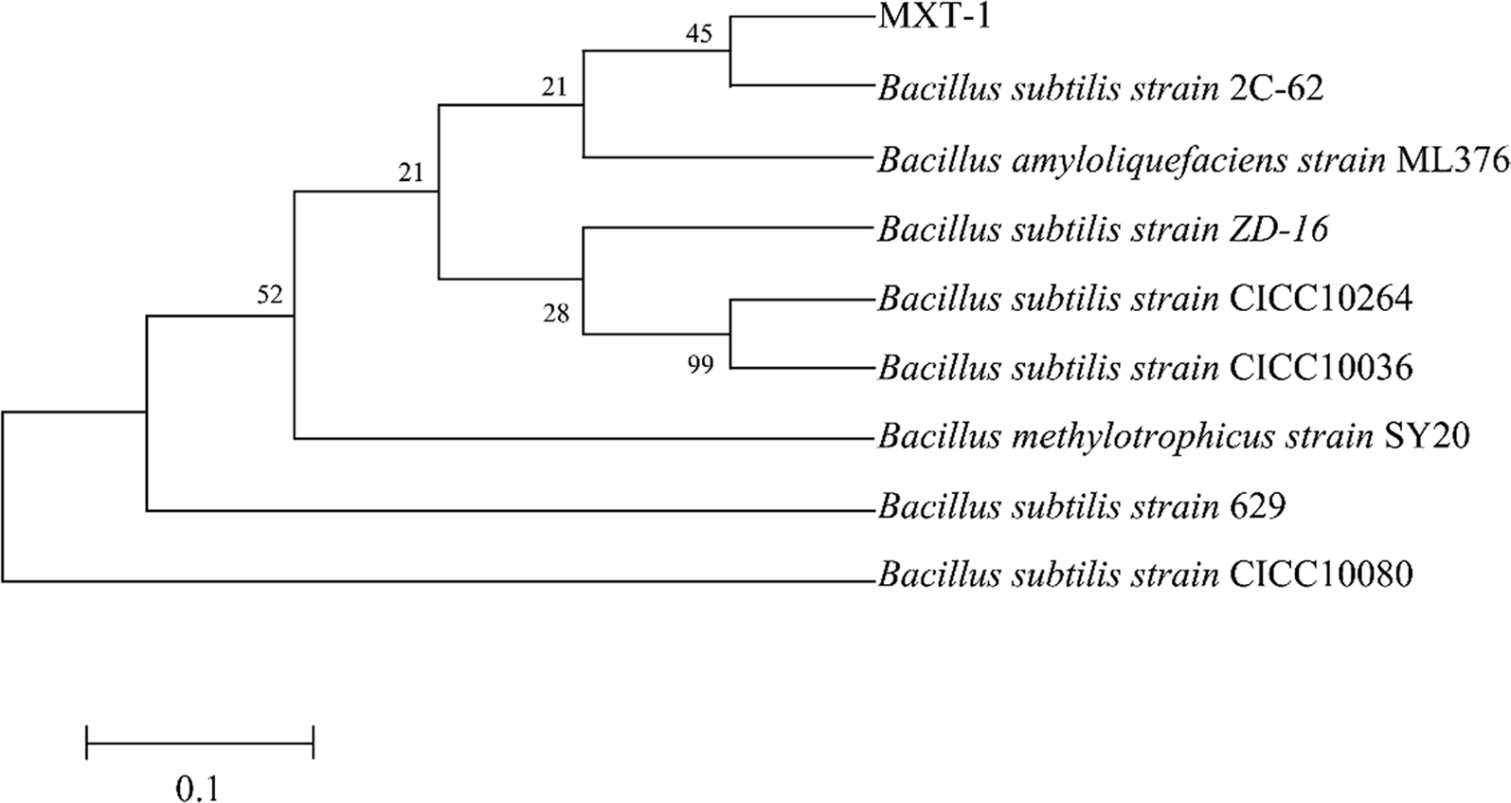
Phylogenetic map for the 16S rRNA gene of strain MXT-1

BLAST analysis of the partial 16S rDNA gene was performed against sequences deposited in GenBank. Based on the BLAST analysis of 16S rDNA, a phylogenetic tree was constructed as shown in Fig. 4. The bacterium was named MXT-1.

Our results showed that strain MXT-1 shared a close relationship with *B. subtilis* strain 2C-62. The 16S rDNA gene was 99% homologous. The MXT-1 strain was subjected to comprehensive morphological and molecular biological analyses. Finally, the strain MXT-1 was classified as *B. subtilis*.

The MXT-1 strain selected in this study had high pentosan degradation activity, suggesting important roles in the degradation and efficient utilization of pentosan in nature.

### Wheat pentosan fermentation and molecular weight analysis

**Fig. 5:**
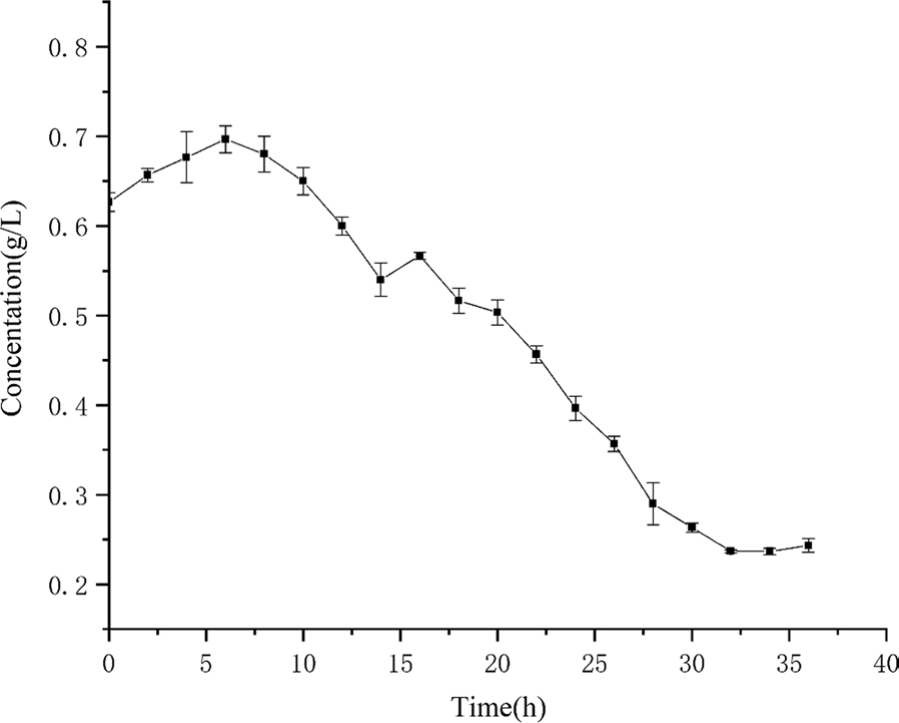
Wheat pentosan concentrations during fermentation

Figure 5 shows changes in total polysaccharides in 36 h of fermentation. Pentosan was slowly degraded by MXT-1 in the initial stage (the first 6 h). Therefore, the decline in the concentration of pentosan was insignificant at the beginning. However, the pentosan concentration decreased rapidly in the intermediate stage (8–32 h) of fermentation, at a rate of about 0.02 g/L per h. The concentration of pentosan decreased from the initial concentration of 0.6 g/L to a final concentration of 0.24 g/L after 36 h of fermentation.

Table 1 shows that the molecular weight of wheat pentosan varied with bacterial-fermentation time. The relative molecular weight of water-insoluble pentosans decreased gradually from 1730 to 257 kDa as the fermentation time increased to 22 h. In this process, the molecular weight of pentosan rapidly dropped by 79% after 14 h of fermentation. However, after 14 h the degradation rate gradually slowed down. The molecular weight of pentosan finally dropped by 85% after 22 h of fermentation. Some of the enzymes produced by the wheat pentosan-degrading bacteria can be secreted extracellularly by the MXT-1 strain. The endo-reaction of β-1,4 glycosidic bonds in wheat pentosan can lead to decreased polymerization. Enzymes acting on the side chains of pentosans may be produced by MXT-1, thereby reducing its branching and increasing its water solubility [20]. Previous findings have shown that the enzymatic degradation of pentosan generally depends on the synergistic effects of arabinofuranosidase and endoxylanase [21].

**Table 1:** Relative molecular weight of wheat pentosan during fermentation

Fermentation time (h)	Relative molecular weight (kDa)
0	1730
12	646
14	360
16	344
18	298
20	270
22	257

### The water-vapor permeability (WVP) of composite films

The WVP of the composite film materials before and after modification was calculated according to Eq. (1). The WVP of the modified composite film decreased from 0.2769 g mm (m^2^ h kPa)^−1^ before modification to 0.1286 g mm (m^2^ h kPa)^−1^. This finding may reflect the better molecular compatibility between wheat pentosan and chitosan and stronger hydrogen bonding between the molecules. Part of the gap in the molecular structure was filled with small pentosan molecules produced by degradation, and the membrane structure became denser. The chain arrangement of hydrogen bonds was enhanced by the flow of free low molecular-weight molecules between longer polymers [22]. These findings are supported by a previous study that showed that the free aldehyde group content in a pentosan solution increases after fermentation, making the film structure more compact [23]. This is because the Maillard reaction between the amino groups of chitosan and the aldehyde groups of pentosan is strong, thus, the amino group content is reduced [23].

### Water solubility of composite films

Water solubility is an important indicator of the stability of biodegradable films. This indicator of the composite film was calculated by Eq. (2). The water solubility of the unfermented pentosan-chitosan composite film was 1.23, whereas that of the modified film was 1.45. Modification increased the number of hydrophilic groups. The lower molecular chains had more water-absorbing active sites than the higher chains. Our results are similar to those of previous studies [22, 24].

### Fourier-transformed infrared (FT-IR) spectroscopy

**Fig. 6:**
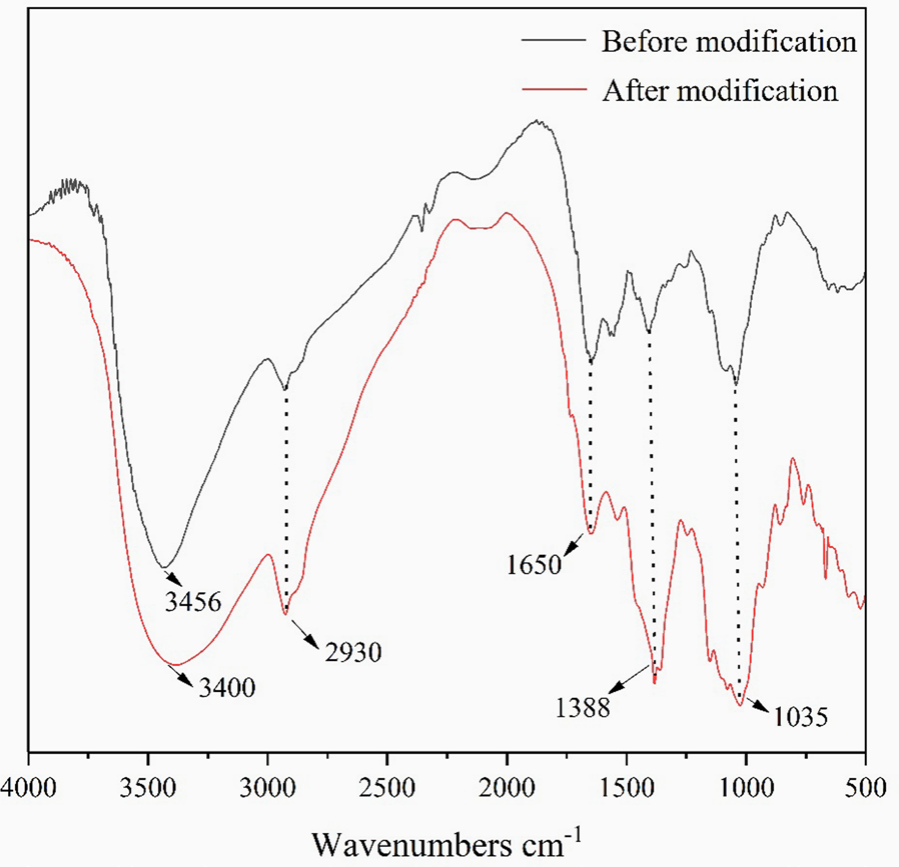
The FT-IR spectra of composite films

Figure 6 shows FT-IR spectroscopy data indicating that wheat pentosan and chitosan molecules interacted before and after modification. Among them, the absorption peak at 3456 cm^−1^ of the unmodified composite film and the absorption peak at 3400 cm^−1^ after modification are attributed to O–H stretching vibration. The absorption peaks of the two composite membranes at 2930, 1650, 1388, and 1035 cm^−1^ are attributed to N–H asymmetric and symmetric stretching vibration, C=O stretching vibration, –NH angular deformation, and C–O–C vibration, respectively. After the modification, the hydroxyl-stretching vibration peak wave number of the membrane material decreased, and the peak shape widened. These results indicate that the modified pentosan/chitosan membrane material had stronger intermolecular hydrogen bonds and improved compatibility with polysaccharide molecules [24].

### Film microstructure examination by scanning electron microscopy (SEM)

**Fig. 7:**
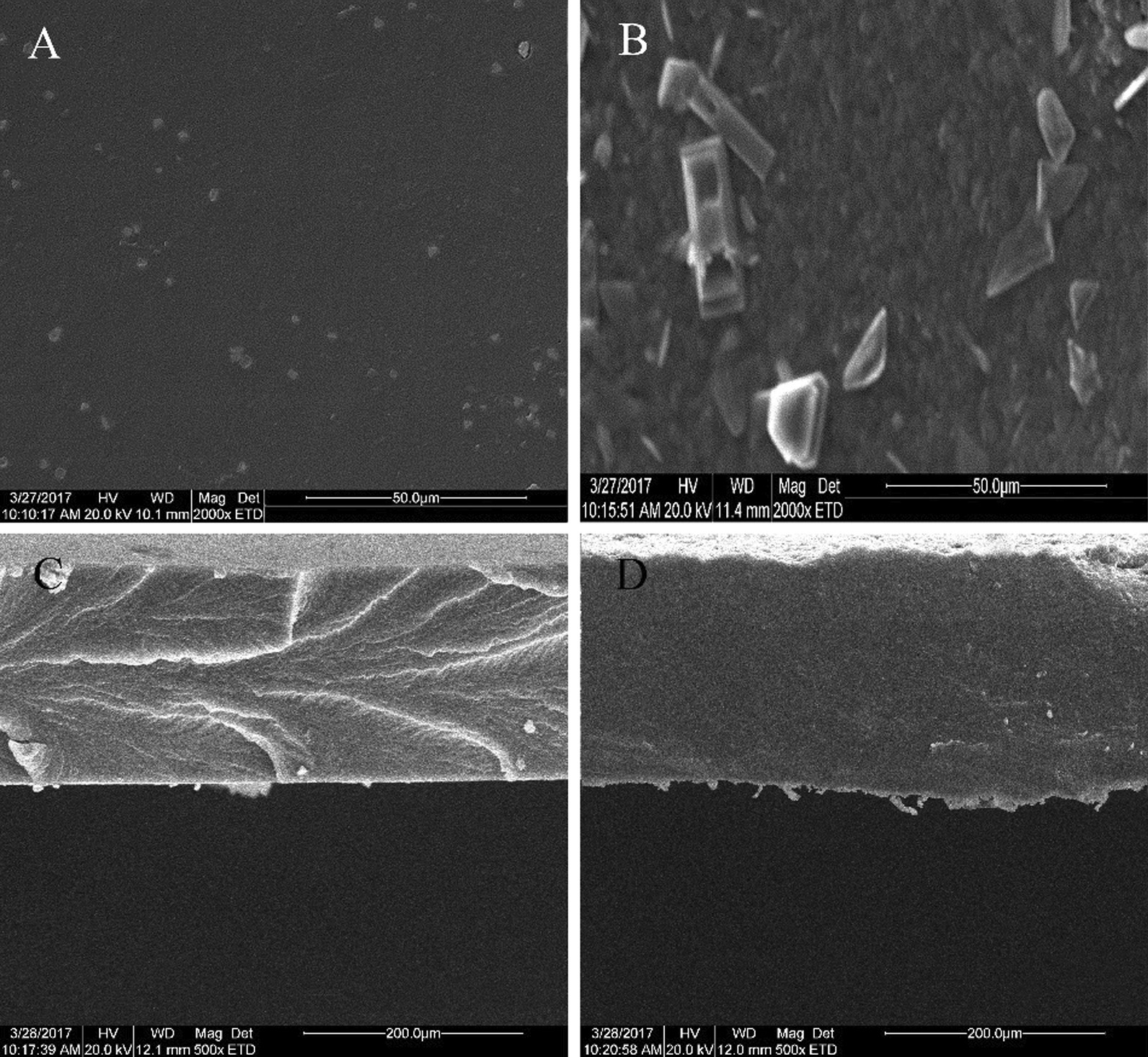
SEM images of composite films. **A**, **C** Surface and cross section of composite film before modification. **B**, **D** Surface and cross section of composite film after modification

The pentosan-modified chitosan surface morphology and cross-section of the composite film were determined by SEM, before and after modification. Figure 7A shows that the surface of the unmodified pentosan/chitosan composite film was smooth, and crystalline particles could only be observed at a magnification of 2000 × . The surface of the film was flat, with no obvious wrinkles or bubbles. The surface of the modified composite film was rougher, which may reflect embrittlement during the liquid nitrogen treatment (Fig. 7B). Figure 7C shows that the cross-sectional thickness of the unmodified composite film was uniform, but the texture was rough. Compared with Fig. 7C, the results in Fig. 7D illustrate that the cross-section thickness of the composite film after modification was uniform, the structure was more compact, and no obvious phase separation occurred. This indicates that it was easier for pentosan to penetrate into the chitosan network structure than before modification, making the hydrogen bond between the components of the films stronger and the connection closer. Similar SEM results were previously obtained for chitosan films containing cellulose and other polysaccharide composite films [24, 25], which also confirmed that the modification increased the compatibility between the pentosan and chitosan molecules.

### Determination of the degree of crystallinity by X-ray diffraction

**Fig. 8:**
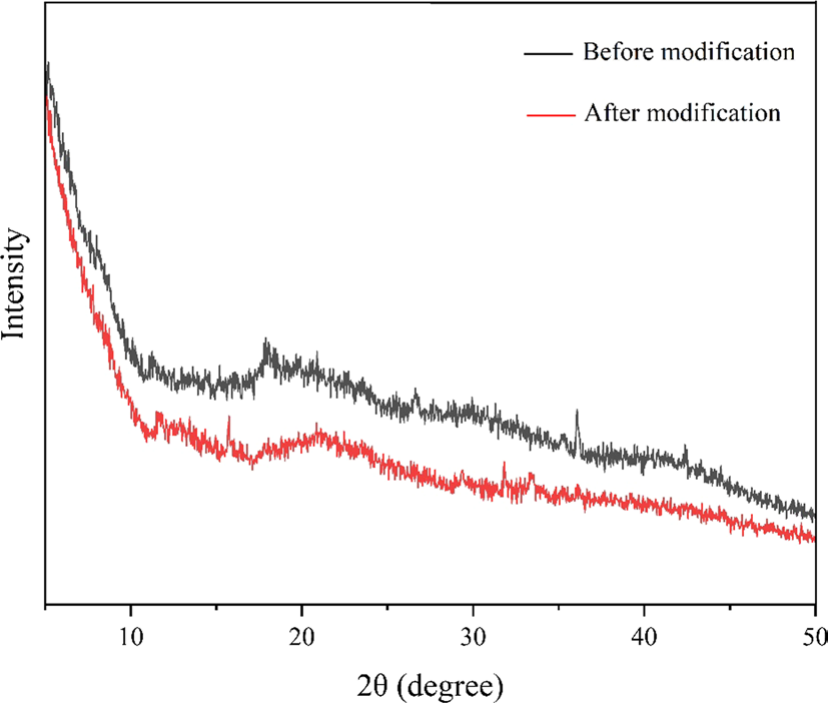
XRD spectra of composite films before and after modification

XRD is a method for analyzing the spatial structures of internal atoms. When the components of a polymer system are compatible, their interactions are strong, which changes their diffraction results. The diffraction curves of the two composite films in Fig. 8 revealed diffraction peaks at 10–20°, although they were not particularly obvious. The 2θ diffraction peaks of the composite film before modification appeared at 15.19° and 17.88°, and the characteristic diffraction peaks of chitosan appeared at approximately 10° and 20°. The 2θ diffraction peaks of the modified composite film were observed at 15.74° and 21.09°. The diffraction peak of chitosan shifts to the higher 2θ direction. Chitosan has a semicrystalline structure [20]. After modification, the compatibility of pentosan and chitosan changed, which interfered with the crystallization. Similar results were reported previously [24].

### Mechanical properties of composite films

Table 2 demonstrates the comparative analysis of the tensile properties of the composite film before and after modification. Before modification, the tensile strength of the composite film was 18.69 MPa, with a maximum displacement of 1.69 mm. Interestingly, the tensile strength of the composite film reduced after modification and was lower than the unmodified film. The decline in tensile strength was by 95%. However, the tensile displacement of the composite film after modification increased significantly. Thus, owing to the increased number of short-chain wheat pentosan molecules in the modified film its flexibility may have increased. The shorter chain pentosan forms additional entanglements between the chains, resulting in the decrease in tensile strength. In addition, low-molecular-weight molecules enhance the mobility of pentosan and chitosan molecules. Previous studies have also drawn similar conclusions [22, 26]. This increased flexibility is extremely beneficial and useful for food packaging films.

**Table 2:** Tensile stress-displacement of the pentosan chitosan composite films

Project	Stress (MPa)	Strain (mm/mm)
Nonmodified pentosan chitosan composite films	18.69	1.69
Pentosan chitosan composite films	0.702	13.65

## Conclusions

The physical properties of polysaccharide films can be improved by using strain MXT-1 to ferment and modify wheat pentosan. The enzyme system acting on the side chain of pentosan may be produced by MXT-1. After biological modification, the solubility of pentosan increased, the compatibility between pentosan and chitosan molecules was improved, and hydrogen bonding was strengthened. The pores between the molecules were filled with small molecules produced by pentosan degradation, which improved the barrier properties of the film. The flexibility of the wheat pentosan composite film were improved, and the water vapor transmission rate was reduced. Furthermore, with a decrease in the molecular weight of pentosan, the tensile strength of the film decreased to a certain extent. Considering the health benefits of pentosans, the composite film is expected to become an effective, edible food-preservation film. In the future, in order to develop a new type of smart food cling film with degradability, antibacterial properties, and green environmental protection, we will explore the effect of changing the concentration of pentosan and chitosan solution, the mixing ratio of pentosan and chitosan, and the molecular weight and structure of modified pentosan on the physical properties and antibacterial properties of the film.

## Methods

### Materials

Wheat B starch-wastewater samples were provided by Shandong Qufeng Food Tech Co., Ltd. (Weifang, China). The composition analysis is presented in Table 3. Water-insoluble pentosans were extracted from wheat B starch wastewater according to Tao’s method [27] and were found to have a relative molecular weight of 1730 kDa. 16S RNA primer synthesis and DNA sequencing were performed by Sangon Biotech Co., Ltd. (Shanghai, China). The DN11-Bacterial Genomic DNA Rapid Extraction Kit, PL03-High Purity Plasmid Small Amount Rapid Extraction Kit, DNA polymerase, DNA ligase, and *Escherichia coli* DH5α cells were purchased from Aidlab Biotechnologies Co., Ltd. (Beijing, China). The reagents used in this study were chemically pure. The name and composition of the culture medium involved in the experiment are shown in Table 4. All the medium ingredients were aseptically prepared.

**Table 3:** Wastewater composition analysis

Parameter	Result
Viscosity (mpa s)	36
Solid content (%)	7.0
Glucose (%)	0.01
Protein (%)	0.43
Ash (%)	6.52

**Table 4:** The name and element of the culture medium

Name	Element (g L^−1^)
Separation medium	Peeled potatoes 200, Glucose 20, KH_2_PO_3_, MgSO_4_ 1.5, Agar 15, pH 6.0
Screening medium	KH_2_PO_4_ 2, NH_4_NO_3_ 2, MgSO_4_·7H_2_O 0.2, Yeast extract 5, Wheat pentosan 20, Agar 20, pH 6.0
Fermentation medium	Wheat pentosan 20, Yeast extract 5, KH_2_PO_4_ 2, NH_4_NO_2_ 2, MgSO_4_·7H_2_O 0.2, pH 6.0
LB medium	Yeast Dip Powder 5, Peptone 10, NaCl 10, pH 6.0
Medium with glucose as the sole carbon source	Glucose 20, KH_2_PO_4_ 2, NH_4_NO_3_ 2, MgSO_4_·7H_2_O 0.2, pH 6.0
Medium with wheat pentosan as the sole carbon source	Wheat pentosan 20, KH_2_PO_4_ 2, NH_4_NO_3_ 2, MgSO_4_·7H_2_O 0.2, pH 6.0

### Separation and screening of strains

One milliliter of sample solution was absorbed from the collected wheat B starch-production wastewater sample solution and diluted 10^−3^-, 10^−5^-, or 10^−7^-fold [28]. An appropriate amount of each dilution was inoculated in separation medium and then incubated at 37 °C for 2 d. The single colony on the separation plate was streaked across a solid medium cultivated as described above, and the transparent circle was observed.

We verified the inducibility of wheat pentosan-degrading enzymes produced by the target strain. As shown in Table 4, glucose and pentosan were used as the sole carbon sources for the two media, respectively [29]. In a sterile environment, the selected strains were respectively inoculated into two media and cultured for an appropriate time. After the fermentation, the bacterial cells were washed several times with sterile water and made into a suspension. 200 μL of each prepared suspension was transferred into separate Oxford cuvettes and incubated at 28 °C for 2 d. For the target strain, we observed whether a transparent circle was produced on the medium with non-pentosan as the carbon source to determine whether the pentosan-degrading enzyme produced by the bacteria was inducible. Additionally, we observed whether there was a transparent circle in the fermentation supernatant of the strain to verify whether the pentosan-degrading enzyme was secreted [30].

### Degradation activity of target strain on wheat pentosan

The activity of this strain was compared with that of other wheat pentosan-degrading strains. The screened strains, *B. subtilis* W800n, *B. licheniformis*, *Pichia pastoris* were cultured in LB medium. After 20 h, 200 μL of the same volume of the four cultured bacterial liquids was inoculated into four Oxford cuvettes on the same solid screening plate and incubated at 28 °C for 2 d [30]. The results of culture were observed and compared.

### Bacterial identification of the target strain

The morphology of the strain was observed, and the basic properties of the strain were verified by Gram staining [31]. Genomic DNA was extracted from the strain, and the target gene sequence was amplified by universal 16S primers (Table 5). The genus of the strain was determined by performing Basic Local Alignment Search Tool (BLAST) analysis against sequences deposited in GenBank [32]. A multiple sequence alignment was performed based on the BLAST analysis of 16S rDNA using sequences with diverse homologies originating from different bacteria. A phylogenetic tree was constructed from the sequence alignment data using the MEGA software (version 5.1).

**Table 5:** 16S rDNA primer sequence

Name	Sequence
27F	5′ AGAGTTTGATCCTGGCTCAG 3′
1492R	5′ TACGGCTACCTTGTTACGACTT 3′

### Wheat pentosan fermentation and molecular weight analysis

The strains were inoculated into the fermentation medium, the composition of the medium is shown in Table 4. Samples were taken every 2 h, and the optical density at 600 nm (OD_600_) was measured [33]. Three parallel experiments were performed, and the growth curves of the strains were drawn using Origin 8 based on the experimental results.

The phenol–sulfuric acid method was used to monitor changes in the wheat pentosan content during fermentation [34]. Wheat pentosan exists in water-soluble and non-water-soluble forms. To increase the degradation effect of wheat pentosan, we first cultured the strain in LB medium containing 0.1% glucose until the OD_600_ exceeded 8.0, after which the cells were precipitated, washed, and cultured further on a shaker for 36 h. The pentosan-degradation process was monitored, and the fermentation broth was collected every 2 h. The pentosan content generated during fermentation was analyzed according to the phenol–sulfuric acid method.

The molecular weight of the wheat pentosan was analyzed after fermentation. DEAE-cellulose 52 was used to purify and analyze the fermented wheat pentosan [35]. Sepharose-CL 6B chromatography was used to analyze the relative molecular-weight distribution. The phenol–sulfuric acid method was used to determine the polysaccharide contents in real time [36]. Dextrans with different relative molecular weights were collected, and standard curves were constructed [36]. A Sepharose-CL 6B column was used to analyze the relative molecular weights of the wheat pentosan species after fermentation.

### Preparation of pentosan-chitosan composite films

Wheat pentosan and chitosan were used to prepare composite films. Wheat pentosan was suspended in water and stirred to obtain a wheat pentosan solution. Chitosan was added to a 1% acetic acid solution to prepare a 1.5% chitosan solution. Both solutions were mixed to obtain a mixed film solution [37]. The resulting solution was heated to 60–65 °C, 1% glycerin was added as a plasticizer, and mixing was performed using magnetic stirring for 4 h. The film liquid was degassed by sonication for 10 min, cast onto a glass plate, and naturally dried at 23 °C and 50% relative humidity (RH). The films were removed from the board for use.

A modified pentosan film solution was obtained after strain fermentation and used to prepare films by mixing biologically modified wheat pentosan with chitosan. The chitosan film solution was prepared according to the method described above. The performance of the two pentosan chitosan composite films before and after modification were analyzed [38].

### The water-vapor permeability (WVP) of composite films

The water-vapor permeability (WVP) of each sample was measured according to American Society for Testing and Materials standard 2005 [39]. Each film was sealed with an aluminum cup containing 43 g dry CaCl_2_, and the cup was placed in an environment with 100% RH. A 6-mm air gap was present between the desiccant and the underside of the film. An aluminum cup was placed in a drying box with a fan. The fan circulated the air above the sample at rotated at a speed of 0.15 m s^−1^. A saturated Mg(NO_3_)_2_ solution was used to maintain the temperature at 22 °C while maintaining the RH at 100%. The cup was weighed several times over a period of 4 d, and the WVP was calculated using Eq. (1).

1 $$WVP = \frac{{(m_{2} - m_{1}) \times L}}{A \times t \times \Delta P}$$

where WVP is the water-vapor permeability; *m*_*1*_ and *m*_*2*_ are the masses of the film, vapor-permeable cup, and CaCl_2_ before and after water absorption, respectively; *L* is the thickness of the film; *A* is the effective measurement area; *t* is the measurement interval; and *∆P* is the vapor-pressure difference on both sides of the film.

### Water solubility of composite films

The water solubility of the films was tested as follows. Each dried film was cut into a size of 3 × 3 cm^2^. An analytical balance was used to weigh the mass of each film three times in parallel, and the average value was recorded as m_1_. The composite film was immersed in 50 mL distilled water for 24 h at 25 °C until swelling equilibrium was reached. Filter paper was used to absorb water from the surface of the film material [40]. The mass was weighed three times, the average value of the swollen mass was recorded as m_2_, and the water solubility was calculated according to Eq. (2) [41].

2 $${\text{Water}}\;{\text{solubility}} = \frac{{m_{2} - m_{1} }}{{m_{1} }}$$

where *m*_*1*_ and *m*_*2*_ are the masses of the film before and after swelling, respectively.

### Fourier-transformed infrared (FT-IR) spectroscopy

FT-IR spectroscopy is a technique that detects chemical bonds in molecules by generating infrared absorption spectra of solids, liquids, or gases. An infrared spectrometer (IR Prestige-21, Shimadzu) was used to analyze the infrared spectra of the composite films before and after modification [42].

### Film microstructure examination by scanning electron microscopy (SEM)

A Quanta 200 SEM instrument (Field Electron and Ion Company) was used to observe the microstructure of the surface and section of the wheat pentosan-chitosan composite films, before and after biological modification [39].

### Determination of the degree of crystallinity by X-ray diffraction

A D8-ADVANCE XRD instrument (AXS) used to determine the crystallinity of the wheat pentosan films, before and after biological modification [42].

### Mechanical properties of composite films

To determine the tensile strength of the films, the films were cut into strips with a width of 5 mm and length of 100 mm. Each strip of film was cut into three pieces for tensile testing. A universal testing machine (Instron 5943, Instron) was used to measure the mechanical properties at 23 °C and 50% RH. The initial distance between the clamps was 50 mm, and a 0.3 N load cell was used for testing at 5 mm min^−1^. The tensile strength and tensile displacement of the three test pieces were measured, and the average of the parallel test results was calculated [43].

## Data Availability

The datasets generated during and/or analyzed during the current study are available in the *figshare* repository, [http://dx.doi.org/10.6084/m9.figshare.16782541].
